# Einthoven dissertation prizes 2014

**DOI:** 10.1007/s12471-015-0697-2

**Published:** 2015-04-21

**Authors:** E.E. van der Wall, J.W. Deckers, V.A.W.M. Umans

**Affiliations:** 1 Netherlands Society of Cardiology/Holland Heart House, Moreelsepark 1, 3511 EP Utrecht, The Netherlands; 2 Department of Cardiology, Erasmus MC - University Medical Center Rotterdam, PO Box 2040, 3000 CA Rotterdam, The Netherlands; 3 Department of Cardiology, Medical Center Alkmaar, Alkmaar, The Netherlands

## Abstract

For the 26th time in a row the Interuniversity Cardiology Institute of the Netherlands (ICIN-Netherlands Heart Institute) and the Netherlands Society of Cardiology (NVVC) have supported the competition for the best three cardiovascular PhD theses, published in the year 2014 [1–3]. The dissertation prize carries the name of one of the greatest Dutchmen in the history of cardiovascular medicine, Willem Einthoven, who in 1902 for the first time recorded the human ECG, for which he received the Nobel Prize in 1924 [4].

This time the jury received a total of 28 PhD dissertations published in 2014. The jury members were very much impressed by the high scientific quality of the PhD fellows. The ultimate selection was based on a combination of several parameters: the curriculum vitae of the candidate, the scientific originality of the PhD thesis and its relevance for the cardiovascular field. In addition, several objective bibliometric parameters were used: (1) the number of articles in first-rate journals both in PubMed and the Web of Science (WOS), (2) the number of citations in WOS, (3) the Hirsch index and (4) the contribution as a first author (or shared first author).

Based on a combination of these results, the jury finally selected three nominees: K.Y. van Spaendonck-Zwarts (University Medical Centre Groningen), N.M. van Mieghem (Erasmus Medical Centre, Rotterdam) and W.J. Dewilde (Sint Antonius Hospital, Nieuwegein).

The members of the jury were: J.W. Deckers (Director CVOI), S. Heymans (ICIN professor), A. Mosterd (Chairman WCN), M.J. Schalij (Chairman Concilium NVVC) and V.A. Umans (President NVVC).

The three candidates presented their Ph.D. theses at the annual spring meeting of the NVVC, held at the Congress Centre “De Leeuwenhorst” in Noordwijkerhout, 9–10 April 2015. Based on the quality of the presentation, the audience determined the ranking of the laureates. Mrs. dr. K.Y. van Speandonck-Zwarts received the third prize, dr. N.M. van Mieghem the second prize, and dr. W.J. Dewilde the first prize. We like to congratulate the three winners with their excellent PhD Theses. Summaries of the three nominated PhD theses are given below.

## References

1. van der Wall EE, Schalij MJ, Umans V, van Gilst WH. Einthoven dissertation prizes 2011. Neth Heart J. 2012;20:240–4. doi:10.1007/s12471-012-0280-z.

2. van der Wall EE, van Gilst WH, Schalij MJ, Umans V. Einthoven dissertation prizes 2012. Neth Heart J. 2013;21:256–61. doi:10.1007/s12471-013-0404-0.

3. van der Wall EE, Umans VA. Einthoven dissertation prizes 2013. Neth Heart J. 2014;22:249–52. doi:10.1007/s12471-014-0555-7.

4. Umans VA, van der Wall EE. 80 years of Netherlands Society of Cardiology. Neth Heart J. 2014;22:135–6. doi:10.1007/s12471-014-0537-9.

## Summary

### **Inherited cardiomyopathies: genetics and gene-environment interactions**

In the last two decades, there have been significant breakthroughs in unraveling genetic factors underlying inherited cardiomyopathies. This is especially important for the relatives of patients with cardiomyopathies. Timely diagnosis of cardiomyopathies and identifying individuals at risk provide the possibility of preventing complications and reducing morbidity and mortality.

Dilated cardiomyopathy (DCM) is highly heterogeneous and shows clinical and genetic overlap with other cardiomyopathy subtypes. In some cases, it may occur with neuromuscular involvement. An overview of 10 years experience with genetic analysis in a large well-defined cohort of idiopathic DCM patients (*n* = 418) shows that clinical clues, such as associated neuromuscular disease or signs of familial occurrence, can strongly increase the yield of genetic analysis in DCM. A *PLN* founder mutation and *LMNA* mutations were most prevalent and often demonstrated a specific phenotype. One of the numerous (> 50) known DCM-related genes is the *DES* gene. *DES* mutations cause desmin-related myopathy, an inherited skeletal and cardiac myopathy. A meta-analysis of reported *DES* mutation carriers (*n* = 159 with 40 different mutations) showed that 50 % of carriers exhibited both neurological and cardiological signs, and strikingly nearly a quarter of carriers exhibited isolated cardiological signs. The cardiac phenotype of two founder *DES* mutations (p.S13F and p.N342D) is characterised by cardiomyopathy and cardiac conduction disease and/or arrhythmias. There are indications that ICD therapy is better than pacemaker therapy for *DES* mutation carriers who are in need of a pacemaker. New genomic techniques, such as next-generation sequencing, now make it possible to analyse larger numbers of genes, even the entire exome or genome, quickly and at reasonable cost. This facilitated the discovery of the genetic cause of an autosomal recessive lethal form of cardioskeletal myopathy. Several Dutch families have children who died with this disorder due to a founder *MYL2* mutation (c.403–1G > C).

Increasing the knowledge on gene-environment interactions is critically important for clinical practice. We have shown that a subset of cases with peripartum cardiomyopathy (PPCM) is part of familial DCM, whereas previously PPCM was classified as a non-genetic, non-familial form of DCM. Hence cardiological screening for covert DCM in first-degree relatives of PPCM patients is advisable, and cardiological screening during pregnancy and puerperium should be considered for first-degree relatives (or relatives carrying an underlying mutation) of familial DCM patients. Targeted next-generation sequencing demonstrated that mutations in cardiomyopathy-related genes, especially in *TTN*, are common in families with both PPCM and DCM. Full and larger-scale analysis of this gigantic gene only recently became available through next-generation sequencing. Presence of an underlying mutation or positive family history for cardiomyopathy in a PPCM patient may be a prognostic factor for low recovery rate. A second hitherto unknown gene-environment interaction in inherited cardiomyopathies is chemotherapy. We have shown that familial predisposition for DCM can be a risk factor for anthracycline-associated cardiomyopathy (AACM). Five DCM families with one AACM patient, and one AACM patient with family members with previously unrecognised early signs of DCM were identified. Moreover, in two of these six families we confirmed the genetic nature of the disease by identifying pathogenic *MYH7* mutations. Thus, prior to treatment of a cancer patient with anthracyclines, careful evaluation of the family history for cardiomyopathy is advisable. The two examples above show highly penetrant genetic factors that are influenced by environmental factors. If we extrapolate this for less penetrant genetic factors, we can speculate that these play a role in pathomechanisms of numerous, higher prevalent cardiovascular diseases.

*K. van Spaendonck-Zwarts*

*Academic Medical Centre Amsterdam*

*Email: k.y.vanspaendonck@amc.uva.nl*

## Summary

### **Transcatheter aortic valve implantation-from cutting edge to mainstream**

Over the last decade, transcatheter aortic valve implantation (TAVI) has become an established treatment for selected patients with symptomatic severe aortic stenosis. The scope of this PhD thesis was to provide novel insights into (1) refined TAVI-tailored risk stratification, (2) importance of uniform reporting, (3) procedural complications and longer-term outcome and (4) strategies to improve TAVI results.

Risk stratification for aortic stenosis patients has evolved considerably. The integrated multidisciplinary heart team has replaced conventional surgical models to ultimately assess an individual’s operative risk and determine the best treatment strategy.

Accurate understanding of TAVI-related complications would help push this novel technology forward. The Valve Academic Research Consortium (VARC) was a collaborative effort of academic institutions, research organisations, competent authorities and industry stakeholders and proposed standardised consensus definitions for TAVI-specific clinical endpoints. Its adoption by the clinical community allowed a more reasonable comparison of reported clinical data. The bulk of major vascular complications after TAVI seem related to artery closure device failure. New closure designs and alternative access strategies could help reduce access site complications. Conduction disorders are frequent after TAVI, especially with self-expanding designs. In approximately half of the cases, conduction abnormalities seem to occur following balloon pre-dilatation prior to the transcatheter valve implantation per se. Furthermore, over half of the patients who received a permanent pacemaker after TAVI were no longer pacemaker dependent after 1 year. Clearly device selection, avoidance of balloon dilatation and a watchful waiting approach may lower pacemaker rates after TAVI. Neurological events after TAVI predominantly occur within the first 24 hours of the procedure and seem directly related to invasive manipulations and suboptimal anticoagulation. Indeed, we analysed the content of embolic protection filters that had been deployed in the extracranial vessels during TAVI and found macroscopic debris in 75 % of all patients. Debris stemmed from the aortic wall or aortic valve in 52 % of cases and thrombotic material was identified in half of the patients. These insights highlight the importance of minimising the instrumentation within the vascular tree during TAVI, improved and more reliable per-procedural anticoagulation and potential benefits of cerebral embolic protection devices.

Multi-centre registry data confirmed the impact of learning curve and growing proficiency as TAVI was performed with gradually less major vascular and life-threatening bleeding complications and with improved 1-year survival. Importantly, similar clinical outcome was achieved with different transcatheter valve platforms.

In conclusion, this thesis dove into the specifics of patient selection and clinical outcome with TAVI and has reached out to novel developments that could further improve its practice. Refinements are essential for TAVI to become a safe and durable alternative to surgical aortic valve replacement in a broader set of patients with lower operative risk and preserved life expectancy.

*N.M. Van Mieghem*

*Interventional cardiology, Thoraxcentrum Erasmus Medical Centre, Rotterdam*

*Email: a.vanhuuksloot@erasmusmc.nl*

## Summary

### **Optimal antiplatelet and anticoagulant therapy in patients with oral anticoagulation and coronary stenting**

Chronic oral anticoagulation (OAC) is necessary in patients with mechanical heart valves and in most patients with atrial fibrillation (AF). A large subgroup of these patients has concomitant coronary artery disease (CAD). In case these patients have to undergo percutaneous coronary intervention (PCI), additional dual antiplatelet treatment with aspirin and a P2Y12 inhibitor becomes indicated to prevent stent thrombosis. In a rapidly ageing community, the number of patients suffering from both AF and CAD is steadily increasing. Up to 2012 these patients were treated with ‘triple therapy’ which is the combination of aspirin, clopidogrel and a vitamin K antagonist (VKA). However, this triple therapy also has its downsides. There is an association with an annual bleeding risk of up to 45 % and bleeding is the Achilles heel of triple therapy because it is associated with an increased mortality risk.

This thesis is based upon the clinical problem in this high-risk patient group (AF and PCI) that we encountered in 2007–2008 when patients had a high bleeding tendency when treated with triple therapy. We set out to decrease the bleeding risk without increasing the risk of stent thrombosis. In this thesis, we provide evidence that suggests that the increased bleeding risk outweighs the efficacy (preventing stent thrombosis, myocardial infarction (MI), stroke and thromboembolism) benefit of triple therapy in these patients and we present a possible new strategy of VKA and a P2Y12 inhibitor alone.

Also, in this thesis we have tried to highlight all aspects of performing PCI in patients with long-term OAC, beginning with the investigation of the optimal peri-procedural regimen (bridging or not?). Furthermore, we paid attention to optimising stent choice (drug eluting or bare metal stent) and investigated a possible interaction in the hepatic cytochrome P450 system of both the metabolism of clopidogrel and acenocoumarol. We conclude with a critical appraisal in which the strengths and the weaknesses of the WOEST trial (What is the Optimal antiplatElet and anticoagulant therapy in patients with oral anticoagulation and coronary StenTing trial) are highlighted and we comment on new evidence backing up the results of the WOEST trial. We address important unanswered questions such as the use of the new direct oral anticoagulants in PCI, the optimal duration of clopidogrel treatment, the use of other P2Y12 inhibitors and whether the optimal treatment after stopping clopidogrel is OAC or the combination of OAC and aspirin.

We conclude and recommend consideration of the only combination (OAC and clopidogrel) that has shown favourable results as compared with the recommended triple therapy in AF patients undergoing PCI.

*W. Dewilde*

*Interventional Cardiology, Amphia Hospital, Breda*

*Email: willemdewilde@yahoo.com*

